# Supplementary material for: The interactome of intact mitochondria by cross-linking mass spectrometry provides evidence for coexisting respiratory supercomplexes
Source: Mol Cell Proteomics. 2017 Dec 8;17(2):216–32. doi: 10.1074/mcp.RA117.000470 (PMC5795388; doi:10.1074/mcp.RA117.000470)
Supplement: Supplemental Data [file supp_RA117.000470_133922_0_supp_23978_fzffwf.zip › spectra_annotation/mito_DR_spectra_annotation/39-1-83-1-1-1.pdf]

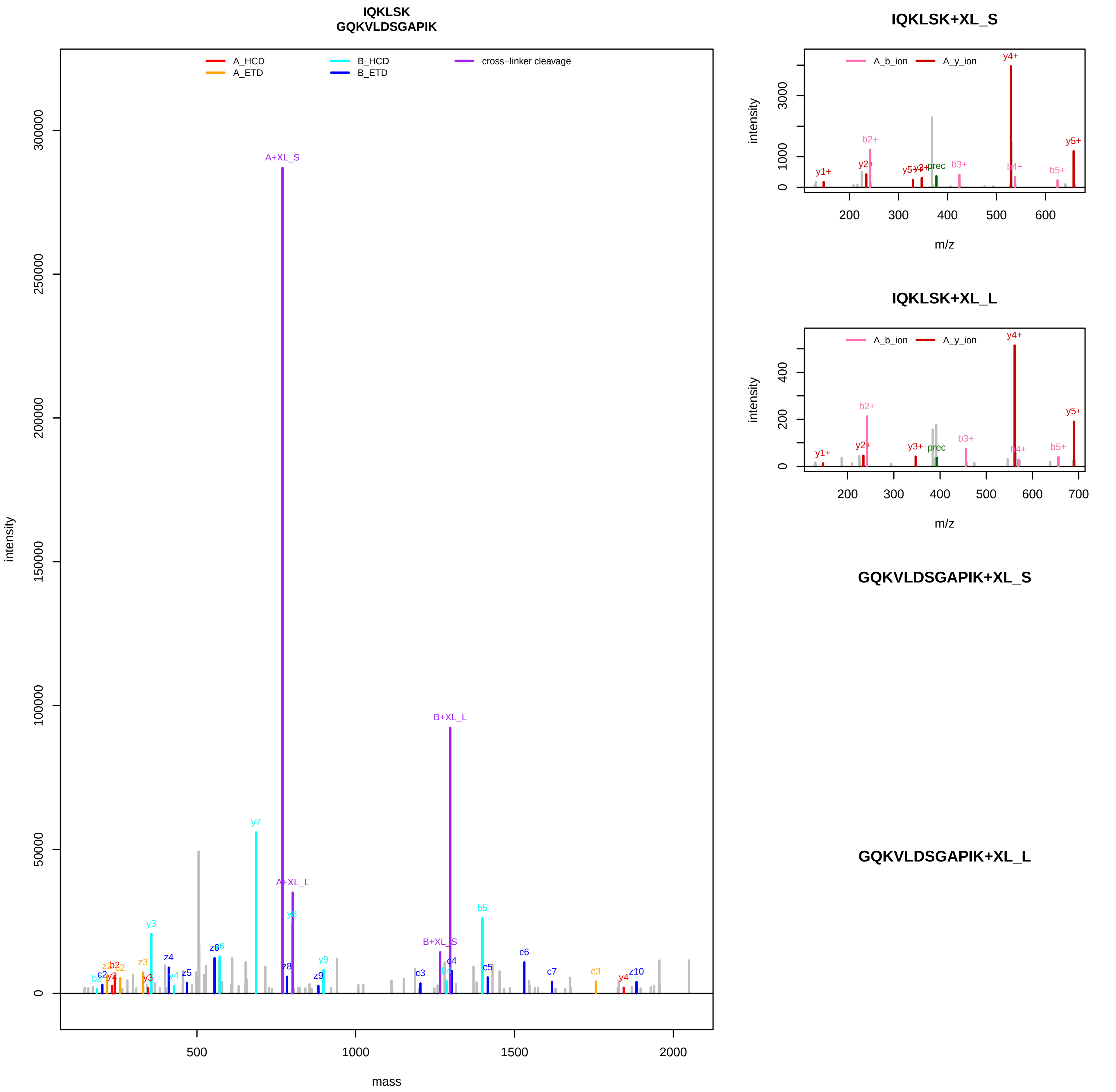

IQKLSK
GQKVLDSGAPIK
A_HCD
A_ETD
B_HCD
B_ETD
cross−linker cleavage
A+XL_S
B+XL_L
A+XL_L
B+XL_S
y7
y3
y8
b5
y9
z4
z5
z6
z8
z9
z10
c2
c3
c4
c5
c6
c7
z3
b2
y2
y3
y4
intensity
mass
0
50000
100000
150000
200000
250000
300000
500
1000
1500
2000
IQKLSK+XL_S
IQKLSK+XL_L
A_b_ion
A_y_ion
y1+
y2+
y3+
y4+
y5+
b2+
b3+
b4+
b5+
prec
m/z
GQKVLDSGAPIK+XL_S
GQKVLDSGAPIK+XL_L
